# Epidemiology and its results in hip fractures followed in postoperative intensive care

**DOI:** 10.1097/MD.0000000000050341

**Published:** 2026-08-28

**Authors:** Emel Yildiz, Murat Emre Tokur, Özlem Arik, Ayşe Yildirimer, Halil Ibrahim Yildiz, Canan Balci

**Affiliations:** a Anesthesiology and Reanimation Clinic, Health Sciences University Evliya Çelebi Education and Research Hospital, Kütahya, Türkiye; b Department of Chest Diseases, Intensive Care Unit, Ege University, İzmir, Türkiye; c Department of Biostatistics and Medical Informatics, Faculty of Medicine, Necmettin Erbakan University, Konya, Türkiye; d Department of Orthopedics and Traumatology, Kütahya Private Park Life Hospital, Kütahya, Türkiye; e Department of Anesthesiology and Reanimation, Faculty of Medicine, Ümraniye Training and Research Hospital, Istanbul, Türkiye.

**Keywords:** APACHE II scores, elderly, hip fracture, intensive care units (ICU), mortality

## Abstract

The global proportion of individuals aged ≥ 65 years is approximately 9%, and hip fractures (HFx) represent one of the most common and serious injuries in this population. This study retrospectively evaluated postoperative intensive care unit (ICU) admissions among patients with HFx, focusing on 30-day, 90-day, and 1-year mortality outcomes. This retrospective study included patients with HFx who were admitted to the ICU after surgery. Collected data included demographic characteristics (age and gender), admission diagnosis, comorbidities, ICU length of stay (LOS), Acute Physiology and Chronic Health Evaluation (APACHE) II scores, American Society of Anesthesiologists score, LOS, 30-day/ 90-day/1-year mortality, day of surgery, and the type anesthesia used. The mortality rate were 34.4% at 30-days 41.3% at 90 days and 24.1% at 1 years after surgery. Patients who died had significantly higher mean age, Acute Physiology and Chronic Health Evaluation II scores, ICU LOS, and total hospital stay. Our findings suggest that postoperative ICU admission criteria for patients with HFx should be reconsidered. The results also emphasize the importance of optimizing ICU LOS in this patient group. Further prospective studies are needed to clarify whether prolonged ICU stay contributes to increased mortality or reflects greater baseline severity.

## 1. Introduction

With advances in healthcare services, technological progress, and increasing life expectancy, the prevalence of age-related diseases has risen substantially. Individuals aged 65 to 74 years are classified as “young old”, 75 to 84 years as “middle old”, and ≥85 years as “advanced age.”^[1]^ Globally, approximately 9% of the population is aged 65 years or older, and this number – estimated at 962 million in 2017 – is projected to exceed 2.1 billion by 2050.^[2]^

Hip fractures are among the most common and debilitating injuries in older adults, representing a major cause of morbidity and mortality. Their etiology is multifactorial, including osteoporosis, sarcopenia, impaired balance, gait instability, and sensory decline.^[3]^ Elderly patients also frequently present with comorbidities such as diabetes mellitus, hypertension, coronary artery disease, and cerebrovascular disorders, which increase perioperative risk and complicate postoperative care.

Although the necessity of postoperative intensive care unit (ICU) admission in elderly hip fracture patients remains debated, previous studies indicate that those with significant comorbidities or perioperative instability may benefit from postoperative ICU monitoring.^[4–6]^ However, evidence evaluating postoperative ICU outcomes in this population is limited.^[5]^

Delays in surgical intervention – often related to ICU bed availability – have been associated with increased morbidity, prolonged hospitalization, and higher mortality.^[7]^ Additionally, several factors, including surgical timing, cognitive impairment, comorbidity burden, and nutritional status, have been shown to influence postoperative outcomes in elderly hip fracture patients.^[8–10]^ Frailty, characterized by diminished physiological reserve and increased vulnerability to stressors, has also been recognized as a key predictor of poor outcomes in this population.

Despite these observations, it remains unclear whether a prolonged postoperative ICU stay independently contributes to increased mortality, or whether patients with greater baseline mortality risk inherently require longer ICU monitoring. Clarifying this distinction is essential for accurate interpretation of clinical outcomes.

Therefore, this study retrospectively evaluated 30-day, 90-day, and 1-year mortality rates in elderly hip fracture patients requiring postoperative ICU admission to better define mortality risk and postoperative care needs in this vulnerable population.

## 2. Materials and methods

### 2.1. Study design and setting

This retrospective observational study was conducted in the General Intensive Care Unit-1 (GICU-1) of Kütahya Health Sciences University Evliya Çelebi Training and Research Hospital between April 2019 and April 2020. The study was approved by the Ethics Committee of Kütahya Health Sciences University (date: 20.01.2021, number: 2021/01-23) and performed in accordance with the Declaration of Helsinki.

### 2.2. Data collection

The following variables were recorded:

- **Demographic data:** age, sex.
- **Clinical information:** admission diagnosis and comorbidities (diabetes mellitus, hypertension, immunosuppression, malignancy, trauma, chronic kidney failure, cardiovascular disease).
- **ICU management variables:** length of ICU stay, Acute Physiology and Chronic Health Evaluation (APACHE) II scores, need for noninvasive mechanical ventilation (NIMV) or invasive mechanical ventilation (IMV), use of inotropic or vasopressor support, renal replacement therapy (RRT) or hemodialysis, blood transfusion, presence of central venous catheter (CVC) or arterial catheterization.
- **Operative variables:** day of surgery and type of anesthesia.
- **Outcomes:** 30-day, 90-day, and 1-year mortality.

### 2.3. Statistical analysis

All analyses were performed using IBM Statistical Package for the Social Sciences Statistics version 20. Descriptive statistics were presented as mean ± standard deviation or median with interquartile ranges for continuous variables and as frequency (n, %) for categorical variables. The normality of distribution was tested using the Kolmogorov–Smirnov and Shapiro–Wilk tests.

For comparisons between groups, the Independent-Samples *t*-test was used for normally distributed variables, and the Mann–Whitney *U* test for non-normally distributed variables. Associations between categorical variables were evaluated using cross-tabulations and appropriate statistical tests.

Predictors of mortality were assessed using binary logistic regression and multivariable logistic regression analyses, and odds ratios with 95% confidence intervals (CIs) were calculated. A *P*-value < .05 was considered statistically significant, whereas *P* < .10 was interpreted as borderline significance.

## 3. Results

A total of 68 postoperative hip fracture (HFx) patients were included in the study. Of these, 61.8% were female and 38.2% were male. Nearly all patients (95.6%) had at least one comorbidity. The mean age was 78.52 years (range: 57–97). Regarding anesthesia type, 11.8% received general anesthesia, 85.3% spinal anesthesia, and 2.9% combined spinal–epidural anesthesia (Table 1).

**Table 1:** Demographic data and comorbidities of the patients, anesthesia method.

Age (n = 68)		Mean ± SD	Min–Max
		78.52 ± 9.15	57-97
Variables	Categories	Frequency (n)	Percent (%)
Gender	Male	26	38.2
	Female	42	61.8
	Total	68	100
Comorbidities	Yes	65	95.6
	No	3	4.4
	Total	68	100
AT	General anesthesia	8	11.8
	Spinal anesthesia	58	85.3
	Spinal + epidural anesthesia	2	2.9
	Total	68	100

AT = anesthesia technique, SD = standard deviation.

Among all patients, 97.1% did not require IMV or inotropic support, and 1.5% required noninvasive mechanical ventilation (NIMV). RRT and intermittent hemodialysis (HD) were applied in 2.9% of patients. Blood transfusion was administered to 27.9% of patients. A CVC was absent in 77.9%, while 26.5% had arterial catheterization (Table 2).

**Table 2:** Interventional procedures.

Variables	Categories	Frequency (n)	Percent (%)
IMV	Yes	2	2.9
	No	66	97.1
	Total	68	100
NIMV	Yes	1	1.5
	No	67	98.5
	Total	68	100
Inotrope and vasopressor support	Yes	2	2.9
	No	66	97.1
	Total	68	100
RRT and HD	3 d in a week	2	2.9
	Yes	1	1.5
	No	65	95.6
	Total	68	100
Blood transfusion	Yes	19	27.9
	No	49	72.1
	Total	68	100
CVC	Yes	15	22.1
	No	53	77.9
	Total	68	100
Arterial catheter	Yes	18	26.5
	No	50	73.5
	Total	68	100

CVC = central venous catheter, HD = hemodialysis, IMV = invasive mechanical ventilation, NIMV = noninvasive mechanical ventilation, RRT = renal replacement therapy.

Overall mortality was 42.6%, whereas 57.4% survived (Table 3). Mortality rates at 30 days, 90 days, and 1 year were 34.48%, 41.38%, and 24.14%, respectively (Table 3). Patients who died had significantly higher mean age, Acute Physiology and Chronic Health Evaluation II (APACHE II) scores, ICU length of stay (LOS), and total hospital LOS compared with survivors (Table 4).

**Table 3:** Mortality of the patients and distribution of patients with exitus.

	Frequency (n)	Percent(%)
Alive	39	57.4
Exitus	29	42.6
Total	68	100

	Frequency (n)	Percent(%)
30-d mortality	10	34.5
90-d mortality	12	41.4
1-yr mortality	7	24.1
Total	29	100

**Table 4:** Differences in mortality status of the patient in terms of age, APACHE II score, ICU and hospital stay, the day before the operation.

	Age		APACHE II		ICU duration of stay		At hospital duration of stay		The day before the operation		Gender			
											Male (n, %)		Female (n, %)	
Alive														
N		39		39		39		39		39		14 35.9%		25 64.1%
Mean		76.64		12.56		1.61		10.46		4.49				
Median		78		12.56		1		9		4				
SD		9.36		4.33		1.09		4.38		2.82				
Min		57		4		1		4		1				
Max		92		23		5		28		12				
25%		70		10		1		8		2				
50%		78		12.5		1		9		4				
75%		83		15		2		13		6				
Exitus														
N		29		29		29		29		29		12 41.4%		17 58.6%
Mean		81.06		16.32		4.65		15.20		6.34				
Median		82		16.32		2		13		5				
Max		97		24		68		68		44				
25%		74.5		13.5		1		8		3				
50%		82		16.3		2		13		5				
75%		88.5		21		3		17.5		7				
*P*-value		.048*		.002*		.085**		.044*		.208				

APACHE II = Acute Physiology and Chronic Health Evaluation II, ICU = intensive care unit, SD = standard deviation.

* *P* < .05.

** *P* < .10.

### 3.1. Regression analysis findings

A 1-year increase in age increased the risk of mortality by 1.059 times (95% CI: 0.999–1.122). A 10-year increase in age increased mortality risk approximately 1.8-fold (e^(0.057 × 10) = 1.768).

Similarly, each 1-unit increase in APACHE II score increased mortality risk 1.187 times (95% CI: 1.056–1.336), and a 10-unit increase increased mortality risk approximately 4.2-fold (e^(0.143 × 10) = 4.179).

Each 1-day increase in hospital stay increased mortality risk 1.079 times (95% CI: 0.987–1.179), and a 10-day increase resulted in a 2.1-fold increase (e^(0.076 × 10) = 2.138) (Table 5).

**Table 5:** Results of univariate logistic regression analysis.

Variables		B	SE	Wald	SD	*P*-value	OR	95% confidence interval (CI) for OR	
								Lower limit	Upper limit
Age	0.057	0.030	3.753	1	.053*	1.059	0.999	1.122
Constant	−4.814	2.358	4.169	1	.041	0.008		
APACHE II scores	0.172	0.060	8.174	1	.004*	1.187	1.056	1.336
Constant	−2.775	0.916	9.172	1	.002	0.062		
Length of stay in hospital	0.076	0.046	2.767	1	.096**	1.079	0.987	1.179
Constant	−1.208	0.580	4.339	1	.037	0.299		

APACHE II = Acute Physiology and Chronic Health Evaluation II, B = beta, OR = odds ratio, SD = standart deviation, SE = standart error.

* *P* < .05.

** *P* < .10.

In the multivariable model, age, APACHE II score, and hospital LOS remained statistically significant predictors of mortality. Mortality risk increased 1.084 times with age (95% CI: 1.007–1.167), 1.196 times with APACHE II score (95% CI: 1.046–1.369), and 1.162 times with hospital stay (95% CI: 1.018–1.327) (Table 6).

**Table 6:** Results of multiplelogisticregressionanalysis of therelationship of mortalitywithage, APACHE II, length of hospitalstay.

Variables	B	SE	Wald	df	*P*-value	OR	95% confidence interval (CI) for OR	
							Lower limit	Upper limit
Step 1†								
Age	0.081	0.037	4.641	1	.031*	1.084	1.007	1.167
Apache II scores	0.179	0.069	6.814	1	.009*	1.196	1.046	1.369
Length ofstay inhospital	0.150	0.068	4.928	1	.026*	1.162	1.018	1.327
Constant	−11.075	3.663	9.139	1	.003*	0.000		

APACHE II = Acute Physiology and Chronic Health Evaluation II, B = beta, df = degrees of freedom, OR = odds ratio, SE = standart error.

† Variable(s) entered on step 1: Age, Apache, length of hospital stay.

* *P* < .05.

### 3.2. Other clinical variables

There was no statistically significant association between mortality and the need for inotropic/vasopressor support at the 0.05 significance level, although a borderline significant association existed at the 0.10 level. No significant difference in mortality was found regarding RRT/HD requirement, blood transfusion, CVC use, or arterial catheterization (Table 7).

**Table 7:** Relationship of mortality with inotropic and vasopressor support, RRT and HD requirement, blood transfusion, CVC, arterial catheter.

Variables	Categories	Alive n (%)	Exitus n (%)	Total n (%)	*χ*^2^; *P*-value
Inotrope and vasopressor support	Yes	0	2 (6.9)	2 (2.9)	2.77; .09*
	No	39 (100)	27 (93.1)	66 (97.1)	
	Total	39 (100)	29 (100)	68 (100)	
RRT and HD requirement	3 d in aweek	0	2 (6.9)	2 (2.9)	4.22; .12
	Yes	0	1 (3.4)	1 (1.5)	
	No	39 (100)	26 (89.7)	65 (95.6)	
	Total	39 (100)	29 (100)	68 (100)	
Blood transfusion	Yes	9 (23.1)	10 (34.5)	19 (27.9)	1.07; .30
	No	30 (76.9)	19 (65.5)	49 (72.1)	
	Total	39 (100)	29 (100)	68 (100)	
CVC	Yes	8 (20.5)	7 (24.1)	15 (22.1)	0.12; .72
	No	31 (79.5)	22 (75.9)	53 (77.9)	
	Total	39 (100)	29 (100)	68 (100)	
Arterial catheter	Yes	8 (20.5)	10 (34.5)	18 (26.5)	1.66; .19
	No	31 (79.5)	19 (65.5)	50 (73.5)	
	Total	39 (100)	29 (100)	68 (100)	

CVC = central venous catheter, HD = hemodialysis, RRT = renal replacement therapy.

* *P* < .10.

## 4. Discussion

The global population is experiencing rapid aging due to declining fertility rates and increasing life expectancy. HFx is widely recognized as a major cause of morbidity and mortality in the elderly and is classified as a “fragility fracture.” Although advancing age contributes to poorer outcomes, age alone is not considered an independent prognostic factor; rather, pre-fracture functional status, cognitive impairment, and comorbidities strongly influence mortality in HFx patients.^[4]^

Previous studies have identified several mortality-related factors, including age, sex, fracture type, functional level, American Society of Anesthesiologists score, time to surgery, and anesthetic technique.^[5]^ Consistent with the literature, the incidence of HFx is higher in women. Guerra et al reported a female predominance of 74.4%, similar to the two-to-five-fold female-to-male ratios in prior reports.^[6,7]^ In our cohort, 61.8% of the patients were female, aligning with these findings.

Hip fracture surgery in the elderly is associated with significant postoperative morbidity and mortality, largely due to multiple comorbidities. Reported postoperative complication rates range from 12.5 to 30%, and 30-day mortality varies from 4.5 to 10%, increasing to 14 to 36% by the end of the first year.^[11]^ Therefore, postoperative ICU admission is often required in high-risk elderly patients.

In a four-year analysis excluding ICU-admitted HFx patients, mortality rates were 13% at 30 days, 33% during hospitalization, and 54% at one year.^[12]^ Hospital admission diagnosis and the timing of ICU admission were key prognostic factors, and perioperative ICU admission was considered appropriate for selected patients.^[12]^ Similarly, a study of 199 patients aged ≥ 65 reported a one-year mortality rate of 23.6%, with a female-to-male ratio of 3:1.^[6]^ Many studies have also shown higher mortality rates in men compared with women.^[8,13]^

The first 30 days after HFx surgery represent the most critical period, with mortality ranging from 3.3 to 24% across studies.^[14–16]^ Daugaard et al. reported a 10% 30-day mortality in 38,020 HFx patients.^[15]^ Caretta et al found that surgery within 2 days significantly reduced mortality.^[16]^ Turhan et al. reported a one-year mortality of 21.7% in patients aged > 65 and identified advanced age and prolonged pre- and postoperative hospital intervals as main predictors.^[17]^

In our study, 30-day, 90-day, and one-year mortality rates were 34.48%, 41.38%, and 24.14%, respectively, consistent with previous reports. Yildiz et al found that 30.5% of geriatric ICU admissions involved postoperative femur fractures.^[18]^ Likewise, Coşkun et al reported orthopedic conditions as the most common cause of ICU admission, accounting for 47% of cases.^[19]^ These findings emphasize the high mortality and morbidity burden in elderly HFx patients and highlight the need for close monitoring and timely ICU management.

D-A Eschbach et al reported a mean ICU stay of 2 days and a hospital stay of 13 to 14 days for patients > 60 years old with femur fractures.^[20]^ In our study, ICU and hospital stays were also prolonged in non-survivors compared with survivors (1.6 vs 4.6 days in ICU and 10.4 vs 15.2 days in hospital). Kanar et al reported a mean hospital-to-operation interval of 5.9 ± 2.7 days, similar to the 4.4/6.3 days observed in our study.^[5]^ Their study emphasized that HFx surgeries should not be delayed due to limited ICU capacity.

The choice of anesthesia in HFx surgery varies with patient characteristics, comorbidities, surgeon preference, and institutional resources. Regional anesthesia has been associated with improved pain control, lower rates of delirium, shorter recovery time, and fewer respiratory complications.^[21]^ However, general anesthesia may be preferred in patients with contraindications to regional techniques or significant cardiopulmonary disease. In our cohort, 88.2% of patients underwent regional anesthesia, consistent with previous findings indicating its benefits in elderly populations.

Malnutrition is common among elderly orthopedic patients, with protein–energy malnutrition reported between 30% and 50%.^[22]^ Yildiz et al highlighted the importance of perioperative nutritional support for femoral fracture patients and suggested that appropriate nutritional intervention reduces morbidity and mortality.^[23]^

In 2018, the Scottish Standards of Care for Hip Fracture Patients were introduced to guide optimal management in elderly HFx patients.^[24]^ Considering the rising elderly population in Türkiye, the implementation of similar national guidelines may be beneficial.

Early geriatric assessment, multidisciplinary management, and correction of comorbidities may improve postoperative outcomes. Preoperative optimization – including nutritional evaluation – and postoperative ICU monitoring may be required to reduce mortality and enhance functional recovery.

## 5. Limitations

This study has several limitations. First, it is a retrospective, single-center study; therefore, generalizability is limited. Second, the sample size was relatively small. Larger, prospective, multicenter studies are needed to better define prognostic indicators and mortality determinants in postoperative HFx patients admitted to ICUs.

## 6. Conclusions

Postoperative ICU admission for HFx patients should be carefully evaluated, as not all patients may require intensive care management. Our retrospective findings indicate that mortality and ICU outcomes are strongly influenced by age, APACHE II scores, and hospital LOS. Optimizing perioperative care and ensuring appropriate patient selection for ICU admission may improve clinical outcomes and reduce unnecessary ICU utilization.

**What Is Known**

- HFx in elderly patients are high-risk conditions and may require postoperative ICU follow-up due to comorbidities and increased complication rates.
- Mortality after HFx surgery is known to be elevated, but the criteria for postoperative ICU admission remain unclear.

**What Is New**

This study provides postoperative 30-day, 90-day, and 1-year mortality rates for HFx patients followed in the ICU; notably, 1-year mortality after postoperative ICU admission has rarely been reported in the literature.

Patients who died had significantly higher age, APACHE II scores, ICU length of stay, and hospital length of stay.

These findings highlight the need for anesthesiologists and intensivists to reevaluate and refine the indications for postoperative ICU admission in HFx patients.

## Acknowledgments

The authors thank Emel Yildiz and Halil İbrahim Yildiz for their assistance with proofreading. The authors also acknowledge Canan Balci for serving as a scientific advisor and Ayşe Yildirimer for contributing to data collection.

## Author contributions

**Conceptualization:** Emel Yildiz, Murat Emre Tokur, Özlem Arik, Ayşe Yildirimer, Halil Ibrahim Yildiz, Canan Balci.

**Data curation:** Emel Yildiz, Murat Emre Tokur, Özlem Arik, Ayşe Yildirimer, Halil Ibrahim Yildiz.

**Formal analysis:** Emel Yildiz, Murat Emre Tokur, Özlem Arik, Ayşe Yildirimer.

**Investigation:** Emel Yildiz, Murat Emre Tokur, Özlem Arik.

**Methodology:** Emel Yildiz, Murat Emre Tokur, Özlem Arik.

**Project administration:** Emel Yildiz, Murat Emre Tokur, Özlem Arik.

**Resources:** Emel Yildiz, Murat Emre Tokur, Özlem Arik.

**Software:** Emel Yildiz, Murat Emre Tokur, Özlem Arik.

**Supervision:** Emel Yildiz, Murat Emre Tokur, Özlem Arik, Ayşe Yildirimer, Halil Ibrahim Yildiz.

**Validation:** Emel Yildiz, Murat Emre Tokur, Özlem Arik, Ayşe Yildirimer, Halil Ibrahim Yildiz, Canan Balci.

**Visualization:** Emel Yildiz, Murat Emre Tokur, Özlem Arik, Ayşe Yildirimer, Halil Ibrahim Yildiz, Canan Balci.

**Writing – original draft:** Emel Yildiz, Murat Emre Tokur, Özlem Arik, Ayşe Yildirimer, Halil Ibrahim Yildiz, Canan Balci.

**Writing – review & editing:** Emel Yildiz, Murat Emre Tokur, Ayşe Yildirimer, Halil Ibrahim Yildiz, Canan Balci.
